# Supplementary material for: Regioselective On-Surface Synthesis of [3]Triangulene Graphene Nanoribbons
Source: J Am Chem Soc. 2024 May 30;146(23):15879–86. doi: 10.1021/jacs.4c02386 (PMC11177251; doi:10.1021/jacs.4c02386)
Supplement: Supplementary file 1 — ja4c02386_si_001.pdf [file ja4c02386_si_001.pdf]

Regioselective On-Surface Synthesis of [3]Triangulene Graphene Nanoribbons

Michael C. Daugherty,[†,‡] Peter H. Jacobse,[‡,‡] Jingwei Jiang,[‡,§,‡] Joaquim Jornet-Somoza,[¤,|] Reis Dorit,[†] Ziyi Wang,[‡,§,¶] Jiaming Lu,[‡] Ryan McCurdy,[†] Weichen Tang,[‡,§] Angel Rubio,[¤,|,¦] Steven G. Louie,[‡,§,*] Michael F. Crommie,[‡,§,¶,*] Felix R. Fischer[†,§,¶,◊,*]

[†]Department of Chemistry, University of California, Berkeley, CA 94720, U.S.A.

[‡]Department of Physics, University of California, Berkeley, CA 94720, U.S.A.

[§]Materials Sciences Division, Lawrence Berkeley National Laboratory, Berkeley, CA 94720, U.S.A.

[¤]Nano-Bio Spectroscopy Group and ETSF, Universidad del País Vasco UPV/EHU, E20018 Donostia, Spain

[|]Max Planck Institute for the Structure and Dynamics of Matter, 227fe61 Hamburg, Germany

[¦]Center for Computational Quantum Physics (CCQ), The Flatiron Institute, New York, NY 10010, U.S.A.

[¶]Kavli Energy NanoSciences Institute at the University of California Berkeley and the Lawrence Berkeley National Laboratory, Berkeley, California 94720, U.S.A.

[◊]Bakar Institute of Digital Materials for the Planet, Division of Computing, Data Science, and Society, University of California, Berkeley, CA 94720, U.S.A.

1.	**Materials and Methods**	S2
2.	**Figure S1.** STM topographic images of molecular precursor **1**, *poly*-**1**, and [3]triangulene-GNRs on Au(111).	S4
3.	**Figure S2.** Summary of BRSTM images and constant height d*I*/d*V* maps of [3]triangulene-GNRs.	S5
4.	**Figure S3.** Summary STS data of [3]triangulene-GNRs.	S6
5.	**Figure S4.** Band structure of [3]triangulene-GNRs calculated from DFT-LDA.	S7
6.	**Figure S5.** Band structure of [3]triangulene-GNRs calculated from DFT-GW and DFT-LDA.	S8
7.	**Figure S6.** Molecular orbital energy calculations of fully *cis*-linked [3]triangulene-GNR.	S9
8.	**Figure S7.** The unfolded band structure of [3]triangulene-GNRs calculated from DFT-LDA and an effective TB model.	S10
9.	**Figure S8**. Mechanistic calculations of the TT coupling of molecular precursor **1** on Au(111).	S11
10.	**Figure S9**. Mechanistic calculations of the HH coupling of molecular precursor **1** on Au(111).	S12
11.	**Figure S10**. Optimized geometry of the diradical HH coupling dimer on Au(111).	S13
12.	**Supplementary Discussion 1:** Additional Factors Influencing the Regioselective On-Surface Polymerization	S14
13.	**Figure S11.** ^{1}H NMR (600 MHz, $CDCl_3$) of 9-(4-bromo-2,6-dimethylphenyl)anthracene (2) at 24 °C.	S15
14.	**Figure S12.** ^{13}C {^{1}H} NMR (151 MHz, $CDCl_3$) of 9-(4-bromo-2,6-dimethylphenyl)anthracene (2) at 24 °C.	S16
15.	**Figure S13.** ^{1}H NMR (600 MHz, $CDCl_3$) of 9-bromo-10-(4-bromo-2,6-dimethylphenyl)anthracene (1) at 24 °C.	S17
16.	**Figure S14.** ^{13}C {^{1}H} NMR (151 MHz, $CDCl_3$) of 9-bromo-10-(4-bromo-2,6-dimethylphenyl)anthracene (1) at 24 °C.	S18
17.	**References**	S19

Materials and Methods

Chemical Synthesis

Unless otherwise stated, all manipulations of air- and/or moisture-sensitive compounds were carried out in oven-dried glassware, under an atmosphere of N_2. All solvents and reagents were purchased from Alfa Aesar, Spectrum Chemicals, Acros Organics, TCI America, and Sigma-Aldrich and were used as received unless otherwise noted. Organic solvents were dried by passing through a column of alumina and were degassed by vigorous bubbling of N_2 through the solvent for 20 min. Flash column chromatography was performed on SiliCycle silica gel (particle size 40–63 μm). Thin layer chromatography was carried out using SiliCycle silica gel 60 Å F-254 precoated plates (0.25 mm thick) and visualized by UV absorption. All ^{1}H and ^{13}C NMR spectra were recorded on a Bruker AV-600 spectrometer and are referenced to residual solvent peaks ($CDCl_3$ ^{1}H NMR = 7.26 ppm, ^{13}C NMR = 77.16 ppm; CD_2Cl_2 ^{1}H NMR = 5.32 ppm, ^{13}C NMR = 53.84 ppm). EI mass spectrometry was performed on an AutoSpec Premier (Waters) system in positive ionization mode. MALDI mass spectrometry was performed on a Voyager-DE PRO (Applied Biosystems Voyager System 6322) in positive ionization mode using a matrix of dithranol.

9-(4-bromo-2,6-dimethylphenyl)anthracene (2). A 100 mL flame-dried Schlenk flask was charged with 5-bromo-2-iodo-1,3-dimethylbenzene (310 mg, 1.0 mmol) in anhydrous Et_2O (25 mL) under N_2. The solution was cooled to 0 °C and *n*-BuLi (0.36 mL, 0.90 mmol, 2.5 M in hexanes) was added dropwise. The solution was stirred for 5 min at 0 °C, and anthrone (0.58 g, 3.0 mmol) was added in several portions under N_2. The reaction was stirred for 2 h at 0 °C and then for an additional 10 h at 24° C. The reaction was quenched by the addition of MeOH (10 mL), concentrated on a rotary evaporator, and dried under vacuum. The reaction flask was charged with *p*-toluenesulfonic acid (0.035 g, 0.20 mmol) and toluene (25 mL), and the reaction was stirred for 30 min at 110° C open to air. The yellow suspension was cooled to 24 °C, washed with saturated aqueous $CaCO_3$ (75 mL), and extracted with CH_2Cl_2 (100 mL). The combined organic phases were dried over Na_2SO_4 and concentrated on a rotary evaporator. Column chromatography (SiO_2; hexanes) yielded **2** (0.36 g, 0.33 mmol, 33%) as a colorless solid. ^{1}H NMR (600 MHz, $CDCl_3$) δ = 8.50 (s, 1H), 8.07 (d, *J* = 8.5 Hz, 2H), 7.49–7.35 (m, 2H), 7.44–7.40 (m, 4H), 7.35 (ddd, *J* = 8.8, 6.4, 1.3 Hz, 2H), 1.72 (s, 6H) ppm; ^{13}C{^{1}H} NMR (151 MHz, $CDCl_3$) δ = 140.3, 136.8, 134.2, 131.7, 130.5, 129.6, 128.9, 126.8, 126.1, 125.6, 125.5, 121.6, 20.0 ppm; HRMS (EI-TOF) m/z: $[C_{22}H_{17}Br]^+$, calcd. $[C_{22}H_{17}Br]$ 360.0514; found 360.0509. (Analysis matches reported values.)[1]

9-(4-bromo-2,6-dimethylphenyl)anthracene (1). A 10 mL Schlenk flask was charged with 9-(4-bromo-2,6-dimethylphenyl)anthracene (20 mg, 0.055 mmol) and N-bromosuccinimide (12 mg, 0.066 mmol) in $CHCl_3$ (1.1 ml) under N_2. The reaction mixture was stirred for 2 h at 60 °C and then cooled to 24 °C. The reaction mixture was concentrated on a rotary evaporator, and the crude residue was dissolved in a minimum amount of acetone, precipitated with MeOH, and filtered over a Buchner funnel to yield **1** (10.5 mg, 0.0239 mmol, 43%) as a pale yellow solid. ^{1}H NMR (600 MHz, $CDCl_3$) δ = 8.62 (d, *J* = 8.8 Hz, 2H), 7.60 (ddd, *J* = 8.9, 6.4, 1.4 Hz, 2H), 7.45–7.42 (m, 4H), 7.39 (ddd, *J* = 8.8, 6.3, 1.1 Hz, 2H), 1.70 (s, 6H) ppm; ^{13}C{^{1}H} NMR (151 MHz, $CDCl_3$) δ = 140.1, 136.4, 135.0, 130.7, 130.6, 130.3, 128.4, 127.3, 126.4, 126.0, 123.0, 121.9, 20.0 ppm; HRMS (EI-TOF) m/z: $[C_{22}H_{17}Br_2]^+$, calcd. $[C_{22}H_{17}Br_2]$ 439.9598; found 439.9599.

[3]triangulene-GNR Growth on Au(111) Surfaces. [3]triangulene-GNRs were grown on Au(111)/mica films under UHV conditions. Atomically clean Au(111) surfaces were prepared through iterative Ar^+ sputter/anneal cycles. Submonolayer coverage of **1** on atomically clean Au(111) was obtained by sublimation at crucible temperatures of 323 K using a home-built Knudsen cell evaporator. After deposition, the surface temperature was slowly ramped (≤5 K min^{-1}) to 473 K and held at this temperature for 30 min to induce radical step-growth polymerization and then ramped up slowly (≤5 K min^{-1}) to 523 K and held for 30 min to induce cyclodehydrogenation.

Scanning Tunneling Microscopy and Spectroscopy. All STM experiments were performed using a commercial Createc LT-STM operating at $T = 4$ K using chemically etched tungsten STM tips. dI/dV measurements were recorded using a lock-in amplifier with a modulation frequency of $f = 533$ Hz and a modulation amplitude of $V_{ac} = 1.0–4.0$ mV. dI/dV point spectra and dI/dV maps were recorded at constant height, under open feedback loop conditions.

Calculations. First-principles DFT calculations in the LDA and LSDA approximations for the electronic band structures were implemented using the Quantum Espresso package.[2-3] We used norm-conserving pseudopotentials with a 60 Ry energy cutoff and 0.005 Ry Gaussian broadening in the presentation of the DOS. To ensure the accuracy of our results, a sufficiently large vacuum region of ~15 Å was included in the supercell calculation. All of the dangling bonds at the edge of the carbon skeleton were terminated with H atoms. The structures were fully relaxed until all components of the force were smaller than 0.01 eV/ Å.

Geometry optimizations for reactant, intermediates, and product species of the dimerization of **1** were computed using the all-electron FHI-aims code at the PBE+VdW level and zeroth order regular approximation (ZORA) scalar relativistic correction.[4-6] We used the van der Waals scheme proposed by Tkatchenko et al.[5] The selected Au(111) surface is modeled using ASE code with four layers of 10 by 11 Au atoms and a *fcc* unit cell parameter of 2.885 Å.[7] The final model consists of 35126 electrons distributed over 518 atoms (39 atoms from each mono-bromo adsorbed molecule **1** and 440 Au atoms) in a periodic lattice with a 28 Å vacuum spacing between the Au surface. We use a standard basis set recommended by the FHI-aims authors: tight defaults for C, N, and H atoms, and light defaults set for the Au atoms. This basis set produces 21714 Kohn-Sham orbitals for each spin component. The system geometries are relaxed until reaching a minimum force threshold of 0.01 eV $Å^{-2}$, keeping the unit cell fixed. During the geometry optimization and transition state search the three inner layers are frozen to reduce the computational cost and simulate the bulk behavior of the surface. The minimum energy path (MEP) for each of the proposed mechanisms are obtained using the string method (SM) as implemented in the *aimsChain* code provided by FHI-Aims.[8-9] For each reaction mechanism, the initial and final structures are interpolated to generate six additional intermediates, and the MEP is reconstructed until reaching a residual force smaller than 0.2 eV $Å^{-2}$, which is sufficient for energy barrier evaluation.[5] Computations starting from the biradical of **1** could not converge due to the presence of multiple minima with different spin multiplicities. Hence, we simplified the model by using the monoradical species bearing C–Br bonds on the non-reacting ends.

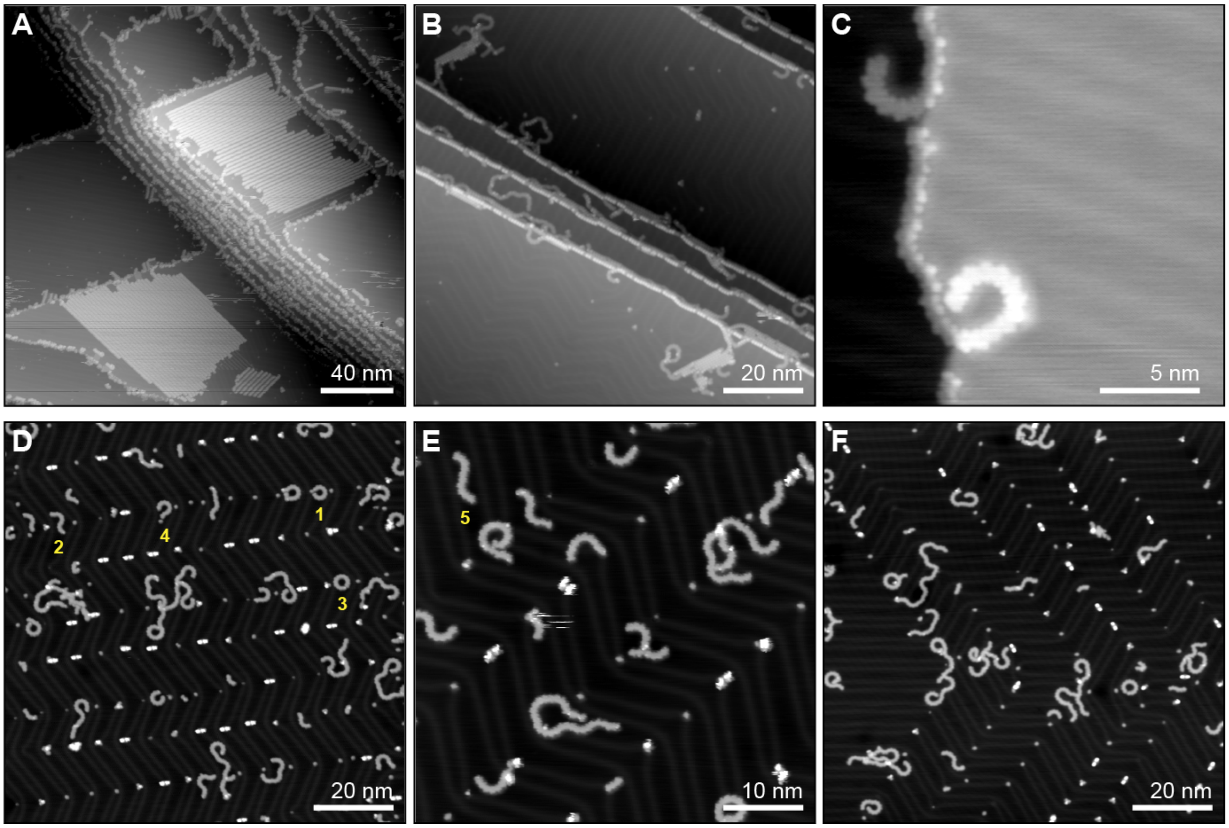

Figure S1. STM topographic images of molecules on Au(111). (A) Self-assembled islands of molecular precursor **1**. (B,C) Chains of *poly*-**1** localized along the Au(111) step edges that have partially cyclodehydrogenated to form [3]triangulene-GNRs following annealing to 200 °C. (D–F) [3]triangulene-GNRs following annealing to 250 °C. A summary of BRSTM and constant height d*I*/d*V* maps for the labeled ribbons (1–5) is presented in Figure S2. A summary of STS data is presented in Figure S3.

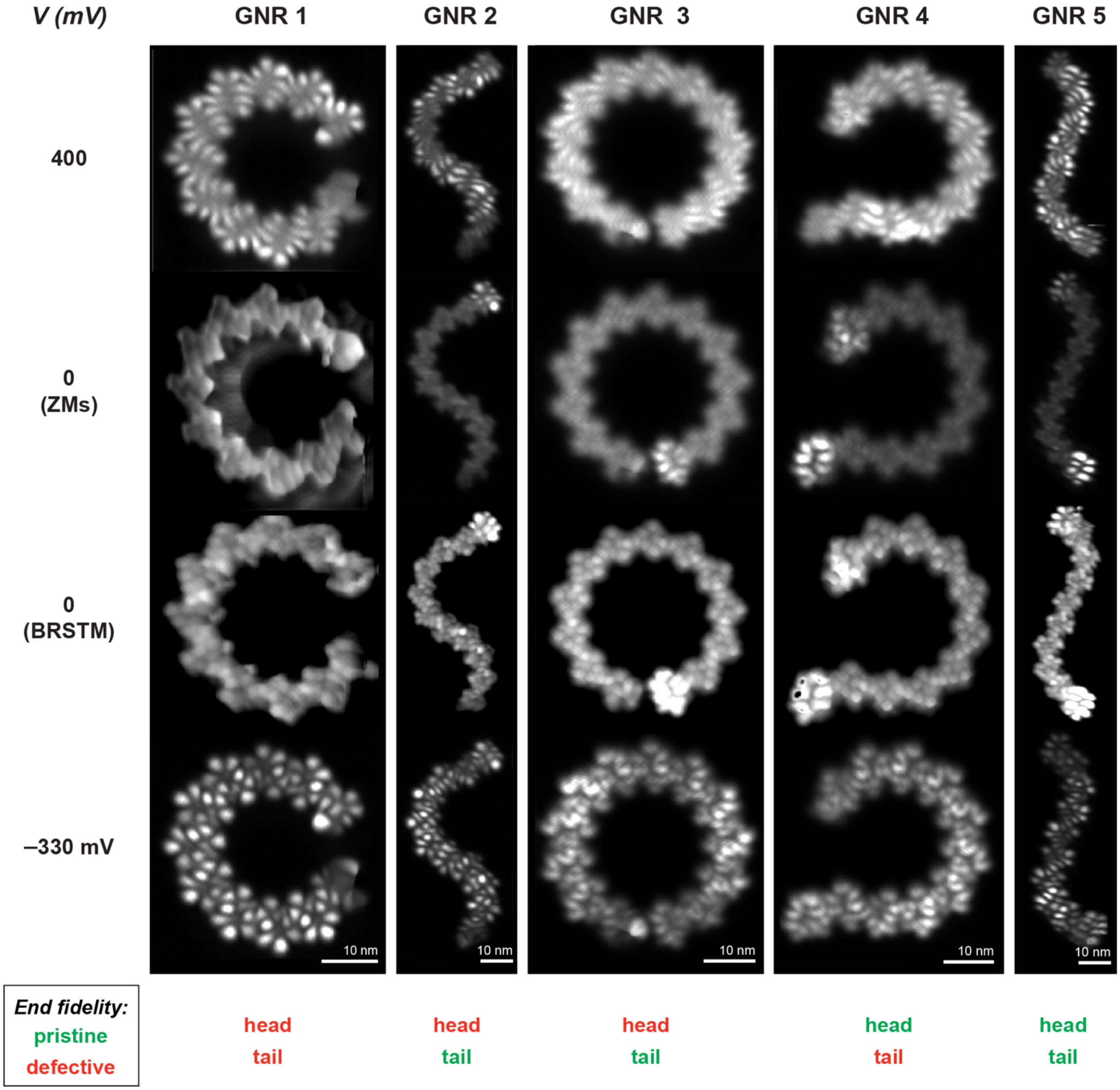

Figure S2. Summary of BRSTM images and constant height dI/dV maps of [3]triangulene-GNRs at the indicated biases. STM topographic images of the GNRs included here are shown in Figure S1D,E. The colored labels indicate whether the [3]triangulene-GNR ends terminated via the head or tail end of molecular precursor **1** (Figure 1A) are pristine (green) or defective (red). The two maps at $V = 0$ differ only in their contrast highlighting either the zero-mode end states or the bonding structure throughout the ribbon.

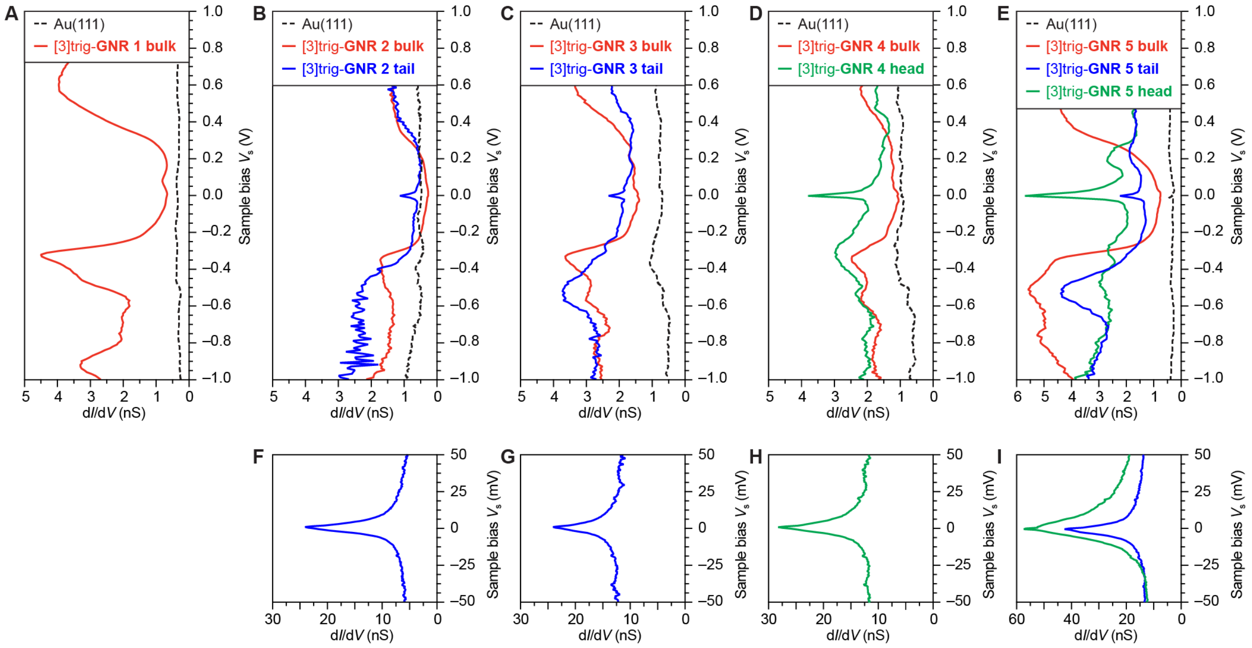

Figure S3. Summary STS data of [3]triangulene-GNRs. (A–E) Constant height STS dI/dV spectra recorded on [3]triangulene-GNRs **1**–**5** (V_{ac} = 0.5 mV, f = 533 Hz). Bulk spectra were recorded at the center of a [3]triangulene unit near the middle of the GNR. (F–I) End state spectra at the head or tail end of the GNR are shown for ribbons with pristine ends. STM topographic images of the GNRs included here are shown in Figure S1D,E.

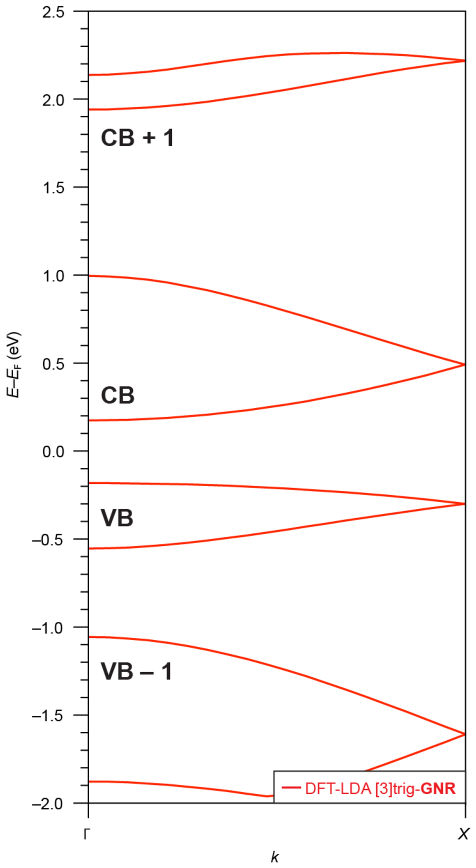

Figure S4. Band structure of [3]triangulene-GNRs calculated from DFT-LDA.

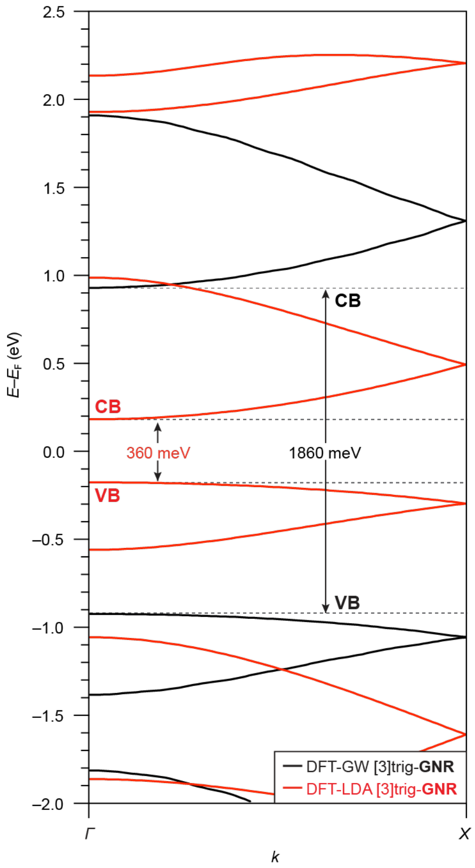

Figure S5. Band structure of [3]triangulene-GNRs calculated from DFT-GW (black) and DFT-LDA (red). The GW calculations were performed using the Berkeley GW package.[10]

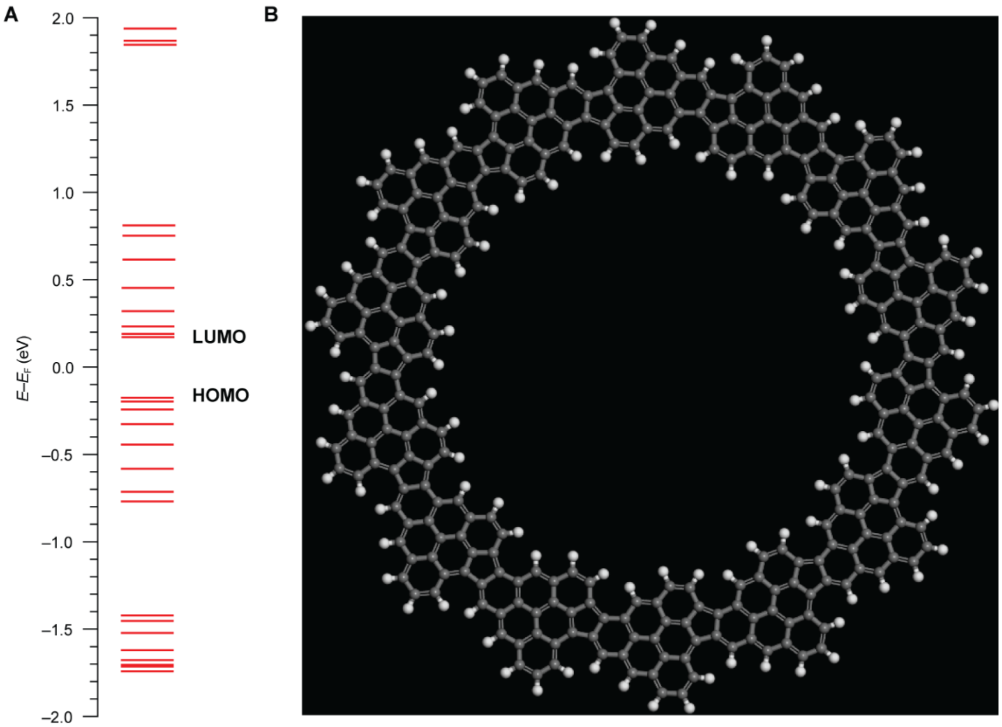

Figure S6 Molecular orbital energy calculations of fully *cis*-linked [3]triangulene-GNR. (A) Molecular orbital energy diagram calculated from DFT-LDA. (B) Fully *cis*-linked cyclic tetradecamer structure used for the calculation.

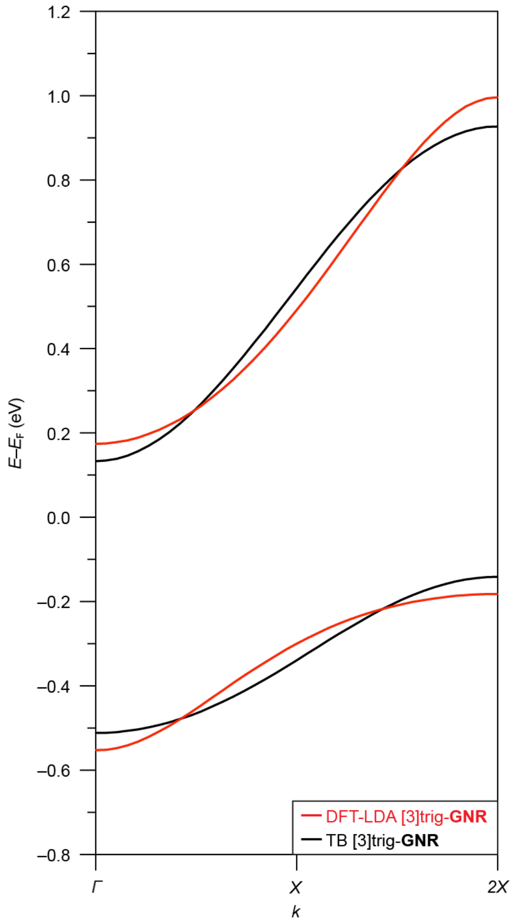

Figure S7. Unfolded band structure of [3]triangulene-GNRs calculated from DFT-LDA (red) and an effective TB model (black).

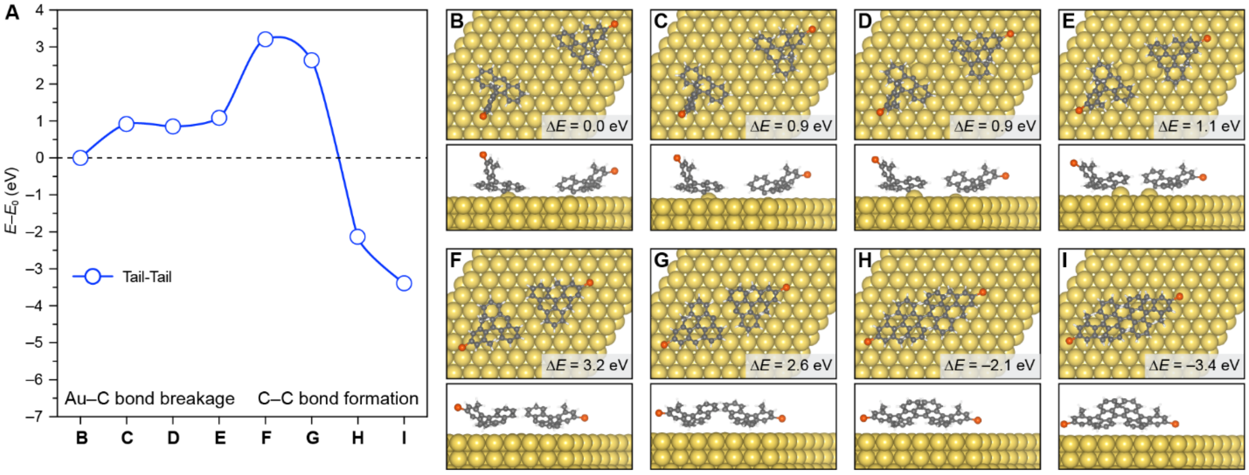

Figure S8. Mechanistic calculations of the coupling of molecular precursor **1** on Au(111). (B) Minimized geometries of molecular precursor **1**, (C–H) intermediates, and (I) the product dimer along the tail-to-tail coupling pathway.

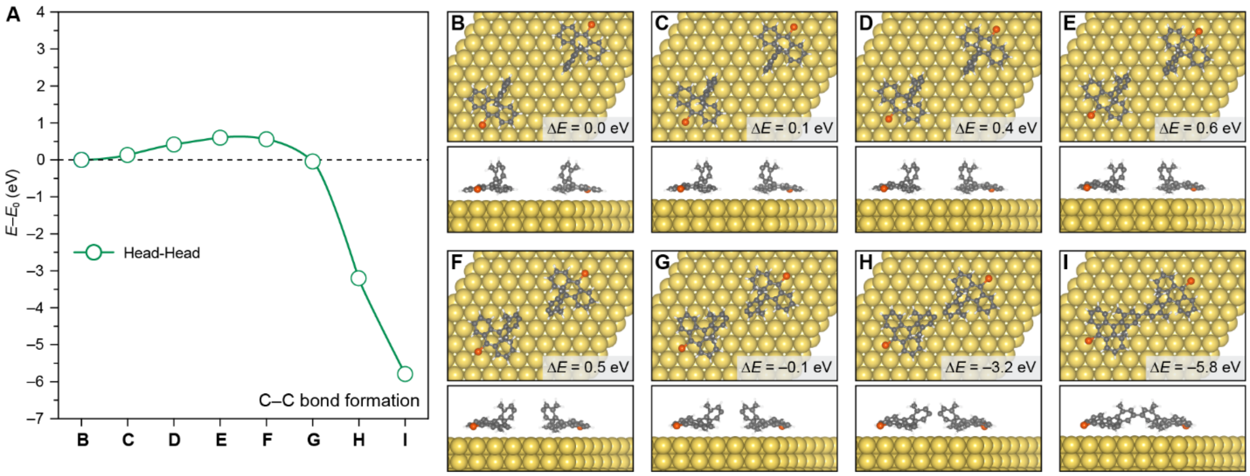

Figure S9. Mechanistic calculations of the coupling of molecular precursor **1** on Au(111). (B) Minimized geometries of molecular precursor **1**, (C–H) intermediates, and (I) the product dimer along the head-to-head coupling pathway.

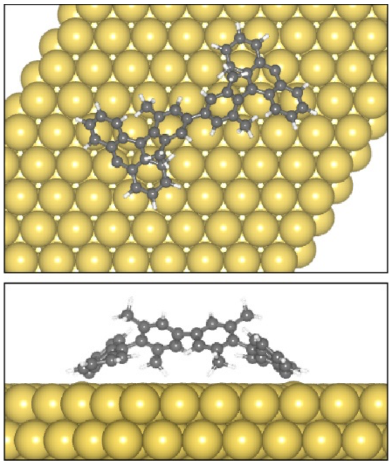

Figure S10. Optimized geometry of the diradical HH coupling dimer on Au(111). The reactive anthryl radical sites are pointing sharply down into the Au(111) surface, likely preventing further coupling.

Supplementary Discussion 1: Additional Factors Influencing the Regioselective On-Surface Polymerization

On-surface reactions are complex and dictated by a diversity of variables. DFT calculations on a Au(111) surface capture factors influencing the polymerization selectivity such as the molecular adsorption geometry and surface reactivity, but other aspects may not be fully accounted for. This includes differences in the bond dissociation energies of the two C–Br bonds of molecular precursor **1**.[11] The 9-anthracenyl C–Br (tail) is weaker, and it likely cleaves first upon annealing. However, TT coupling between the resulting anthracenyl radicals is highly energetically unfavorable. Therefore, the population of this monoradical species may accumulate, and once the temperature is sufficiently high to cleave the phenyl C–Br bond, the newly formed radical reacts preferentially with the excess of anthracenyl radicals. This possible explanation may rationalize why [3]triangulene-GNRs capped with an instance of HH coupling are not observed.

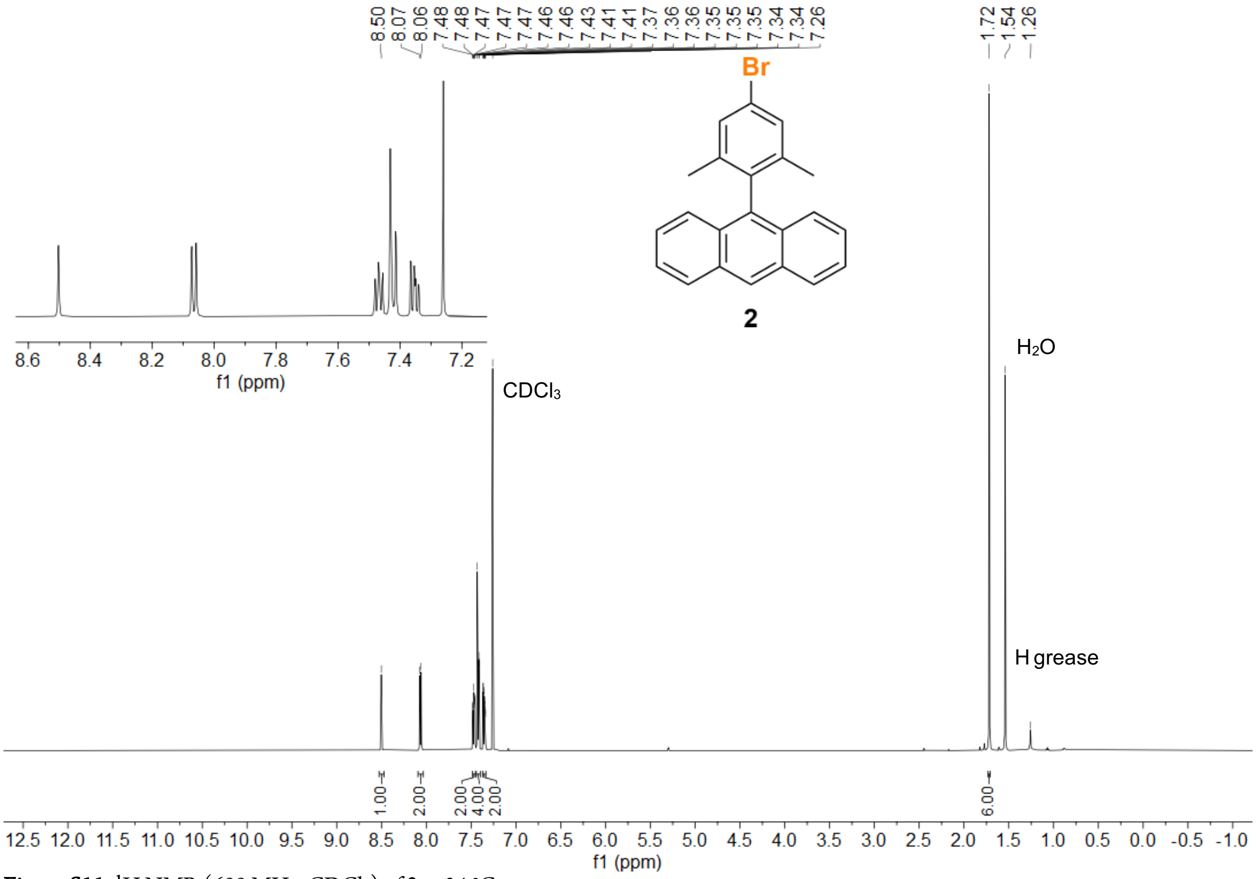

Figure S11. ^{1}H NMR (600 MHz, $CDCl_3$) of **2** at 24 °C.

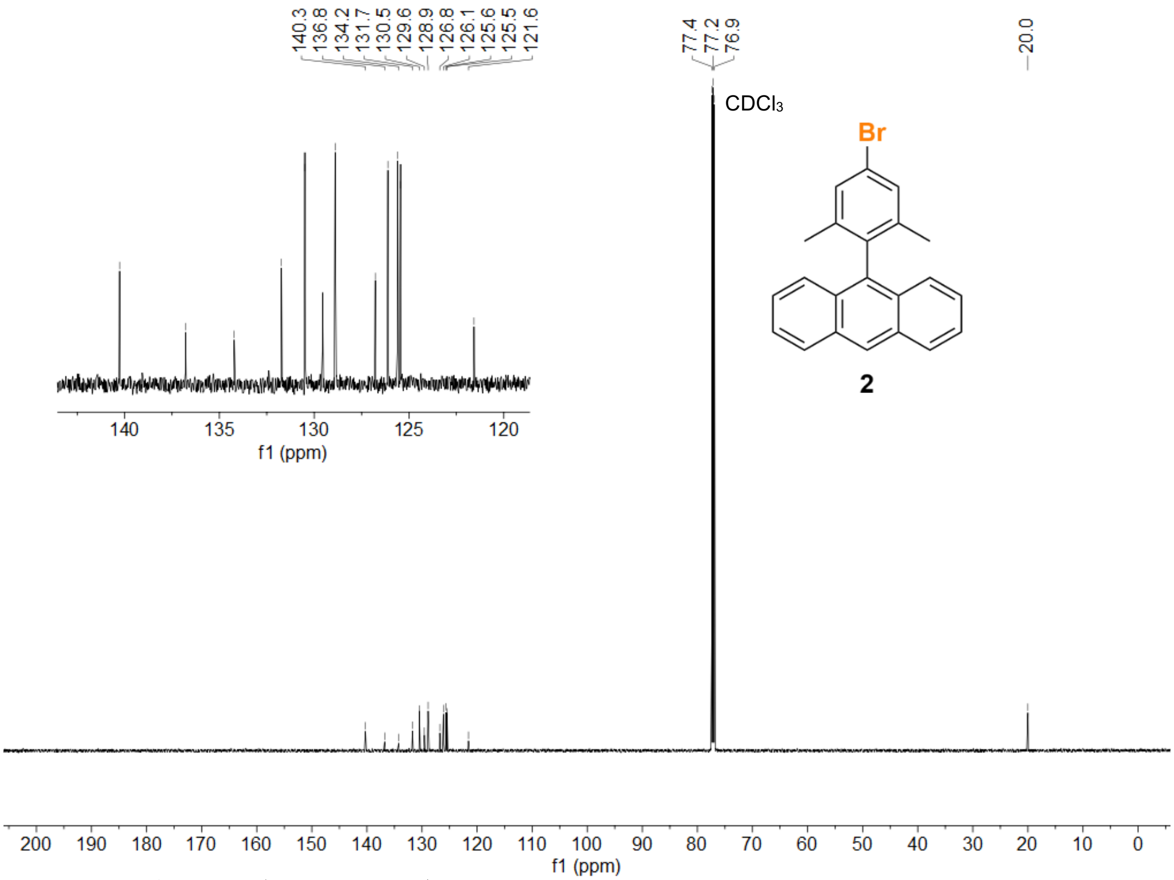

Figure S12. $^{13}C\{^1H\}$ NMR (151 MHz, $CDCl_3$) of **2** at 24 °C.

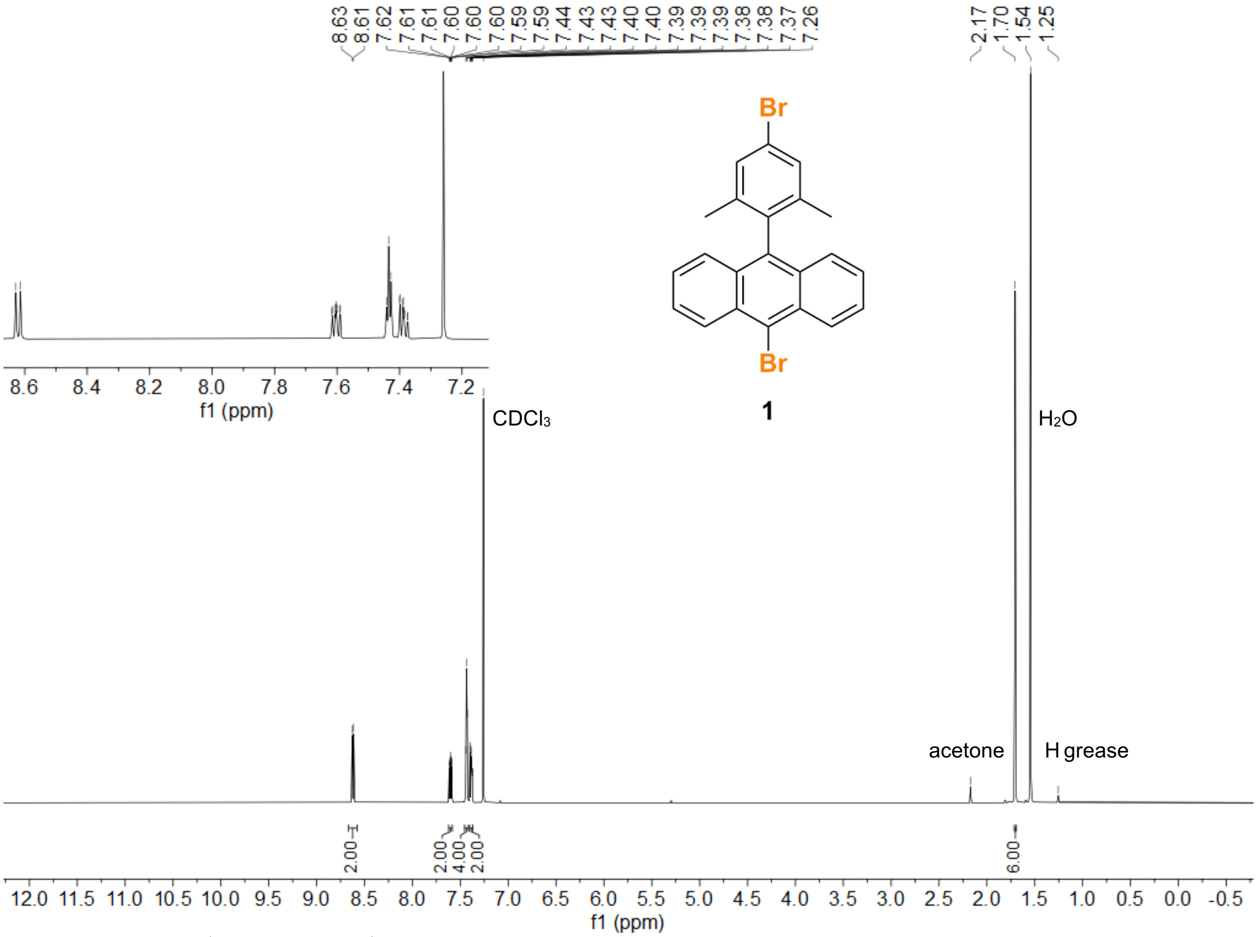

Figure S13. ^{1}H NMR (600 MHz, $CDCl_3$) of **1** at 24 °C.

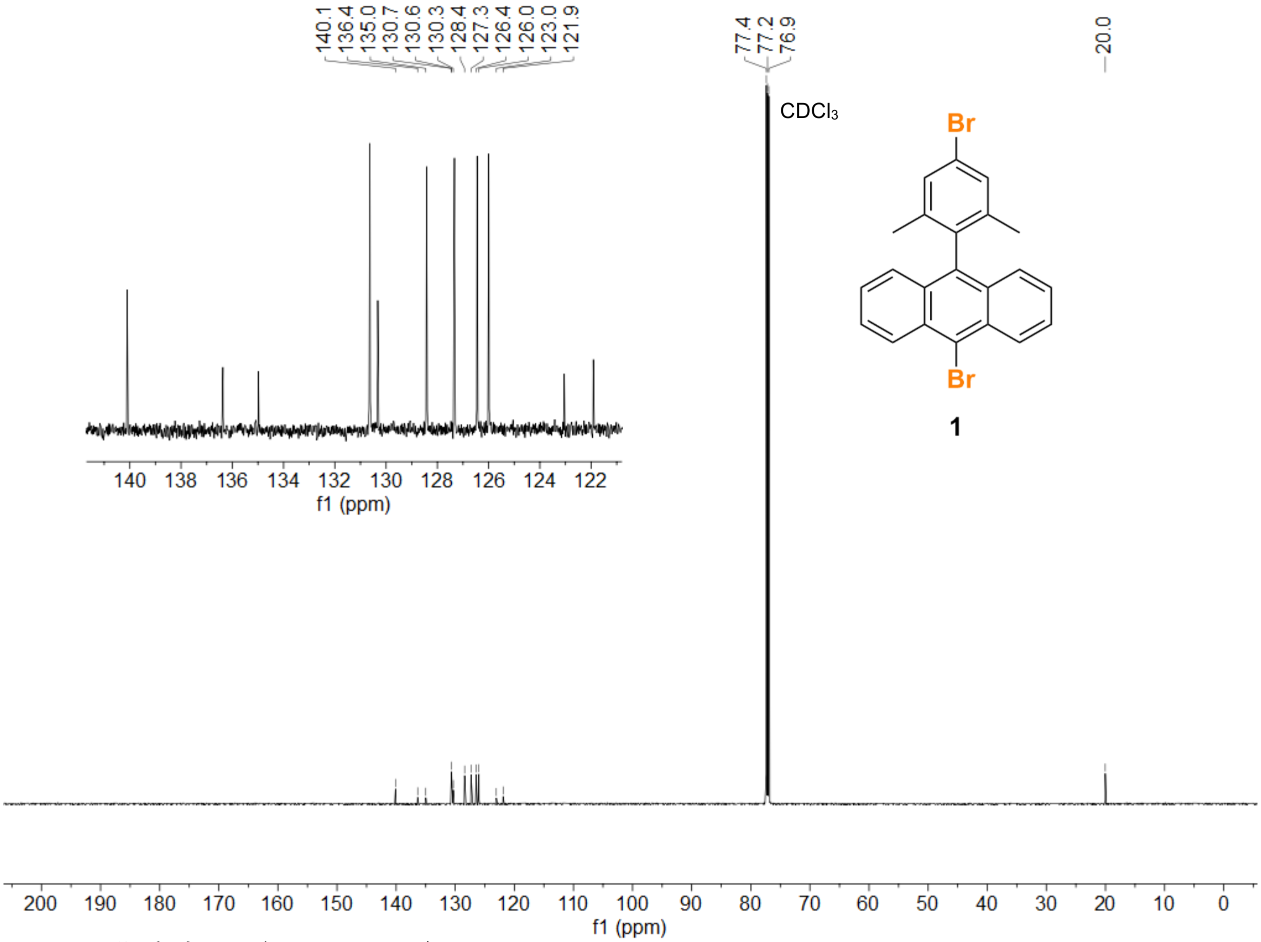

Figure S14. $^{13}C\{^1H\}$ NMR (151 MHz, $CDCl_3$) of **1** at 24 °C.

References

(1) Mishra, S.; Beyer, D.; Eimre, K.; Ortiz, R.; Fernandez-Rossier, J.; Berger, R.; Groening, O.; Pignedoli, C. A.; Fasel, R.; Feng, X. L.; Ruffieux, P., Collective All-Carbon Magnetism in Triangulene Dimers. *Angew. Chem. Int. Ed.* **2020**, *59*, 12041-12047.

(2) Giannozzi, P.; Baroni, S.; Bonini, N.; Calandra, M.; Car, R.; Cavazzoni, C.; Ceresoli, D.; Chiarotti, G. L.; Cococcioni, M.; Dabo, I.; Dal Corso, A.; de Gironcoli, S.; Fabris, S.; Fratesi, G.; Gebauer, R.; Gerstmann, U.; Gougoussis, C.; Kokalj, A.; Lazzeri, M.; Martin-Samos, L.; Marzari, N.; Mauri, F.; Mazzarello, R.; Paolini, S.; Pasquarello, A.; Paulatto, L.; Sbraccia, C.; Scandolo, S.; Sclauzero, G.; Seitsonen, A. P.; Smogunov, A.; Umari, P.; Wentzcovitch, R. M., QUANTUM ESPRESSO: a modular and open-source software project for quantum simulations of materials. *J. Phys. Condens. Mat.* **2009**, *21*, 395502.

(3) Giannozzi, P.; Andreussi, O.; Brumme, T.; Bunau, O.; Nardelli, M. B.; Calandra, M.; Car, R.; Cavazzoni, C.; Ceresoli, D.; Cococcioni, M.; Colonna, N.; Carnimeo, I.; Dal Corso, A.; de Gironcoli, S.; Delugas, P.; DiStasio, R. A.; Ferretti, A.; Floris, A.; Fratesi, G.; Fugallo, G.; Gebauer, R.; Gerstmann, U.; Giustino, F.; Gorni, T.; Jia, J.; Kawamura, M.; Ko, H. Y.; Kokalj, A.; Küçükbenli, E.; Lazzeri, M.; Marsili, M.; Marzari, N.; Mauri, F.; Nguyen, N. L.; Nguyen, H. V.; Otero-de-la-Roza, A.; Paulatto, L.; Poncé, S.; Rocca, D.; Sabatini, R.; Santra, B.; Schlipf, M.; Seitsonen, A. P.; Smogunov, A.; Timrov, I.; Thonhauser, T.; Umari, P.; Vast, N.; Wu, X.; Baroni, S., Advanced capabilities for materials modelling with QUANTUM ESPRESSO. *J Phys-Condens Mat* **2017**, *29*, 465901.

(4) Perdew, J. P.; Burke, K.; Ernzerhof, M., Generalized gradient approximation made simple. *Physical Review Letters* **1996**, *77*, 3865-3868.

(5) Tkatchenko, A.; Scheffler, M., Accurate Molecular Van der Waals Interactions from Ground-State Electron Density and Free-Atom Reference Data. *Physical Review Letters* **2009**, *102*, 073005.

(6) Blum, V.; Gehrke, R.; Hanke, F.; Havu, P.; Havu, V.; Ren, X. G.; Reuter, K.; Scheffler, M., Ab initio molecular simulations with numeric atom-centered orbitals. *Comput Phys Commun* **2009**, *180*, 2175-2196.

(7) Larsen, A. H.; Mortensen, J. J.; Blomqvist, J.; Castelli, I. E.; Christensen, R.; Dulak, M.; Friis, J.; Groves, M. N.; Hammer, B.; Hargus, C.; Hermes, E. D.; Jennings, P. C.; Jensen, P. B.; Kermode, J.; Kitchin, J. R.; Kolsbjerg, E. L.; Kubal, J.; Kaasbjerg, K.; Lysgaard, S.; Maronsson, J. B.; Maxson, T.; Olsen, T.; Pastewka, L.; Peterson, A.; Rostgaard, C.; Schiotz, J.; Schutt, O.; Strange, M.; Thygesen, K. S.; Vegge, T.; Vilhelmsen, L.; Walter, M.; Zeng, Z. H.; Jacobsen, K. W., The atomic simulation environment-a Python library for working with atoms. *J Phys-Condens Mat* **2017**, *29*, 273002.

(8) Weinan, E.; Ren, W. Q.; Vanden-Eijnden, E., String method for the study of rare events. *Physical Review B* **2002**, *66*, 052301.

(9) E, W. N.; Ren, W. Q.; Vanden-Eijnden, E., Simplified and improved string method for computing the minimum energy paths in barrier-crossing events. *J Chem Phys* **2007**, *126*, 164103.

(10) Deslippe, J.; Samsonidze, G.; Strubbe, D. A.; Jain, M.; Cohen, M. L.; Louie, S. G., BerkeleyGW: A massively parallel computer package for the calculation of the quasiparticle and optical properties of materials and nanostructures. *Comput. Phys. Commun.* **2012**, *183*, 1269-1289.

(11) Ladacki, M.; Szwarc, M., Studies of the Variations in Bond Dissociation Energies of Aromatic Compounds .1. Mono-Bromo-Aryles. *Proc R Soc Lon Ser-A* **1953**, *219*, 341-352.
